# Protecting developing brains: urgent need to regulate synthetic food dyes in children’s diets

**DOI:** 10.1097/MS9.0000000000003770

**Published:** 2025-08-28

**Authors:** Sanoober Fatima, Muneeb Saifullah, Maliha Khalid, Muhammad Talha, Aminath Waafira

**Affiliations:** a Dow Medical College, DUHS, Karachi, Pakistan; b King Edward Medical University, Lahore, Pakistan; c Jinnah Sindh Medical University, Karachi, Pakistan; d School of Medicine, The Maldives National University, Malé, Maldives

**Keywords:** ADHD, neurodevelopment, public health policy, synthetic food dyes

## Abstract

Synthetic food dyes remain prevalent in children’s diets despite growing evidence linking them to adverse neurodevelopmental outcomes, including exacerbation of Attention-Deficit Hyperactivity Disorder (ADHD) symptoms. Recent systematic reviews and meta-analyses indicate associations between dye exposure and behavioral changes, with mechanisms involving oxidative stress, mitochondrial dysfunction, and disruption of dopamine and serotonin metabolism leading to neuroinflammation and impaired impulse control. Current safety standards, such as Acceptable Daily Intakes (ADIs), rely on outdated toxicological data and fail to account for cumulative effects or neurobehavioral endpoints. Regulatory inconsistencies, industry resistance, and inadequate labeling hinder effective control. We advocate for updated FDA and EFSA guidelines, reformulation incentives for manufacturers, improved labeling, and public health campaigns promoting natural colorants and additive-free diets. Urgent action is required to protect developing brains and prioritize neurological safety over aesthetic appeal in children’s food products.

*To the Editor,*

The use of synthetic food dyes in food products marketed to children is a growing public-health concern. Recent studies have revealed that synthetic food dyes may exacerbate adverse neurological outcomes in the pediatric population and worsen Attention-Deficit Hyperactivity Disorder (ADHD) symptoms in children. While no certain causative relationship can be established, the emerging data support the risk underlying the use of synthetic food dyes in the neurodevelopment of children. This not only raises concern over the excessive use of food dyes in products marketed to children, but also calls for urgent reassessment of dietary safety standards and efforts to reduce exposure.

A systematic review of 27 clinical trials, conducted by the California Office of Environmental Health Hazard Assessment (OEHHA), reported an association of 64% between synthetic dye exposure and behavioral changes, 52% was statistically significant[1]. A 2012 meta-analysis found that 8% of children with ADHD might have faced symptom exacerbations due to synthetic food dyes[2]. Another review published in 2016 associated food dyes with allergies, asthma, and hyperactivity in children[3], while a meta-analysis of double-blind placebo-controlled trials found a modest but significant association between artificial food colorings and increased hyperactivity in sensitive children with hyperactive syndromes[4].

Food additives contribute significantly to neurological and behavioral changes. A review published in *Chemosphere* (2024) highlighted that dyes such as Tartrazine and Allura Red increase oxidative stress by increasing the generation of Reactive Oxygen Species (ROS), damaging neuronal cells and cell membranes, and causing mitochondrial dysfunction[5]. The report by OEHHA also mentioned that some dyes interfere with Dopamine and Serotonin metabolism and cause neuroinflammation and histamine release, leading to hyperactivity, mood swings, attention deficit, and loss of impulse control[2].

Despite emerging evidence, regulating processed food ingredients remains a challenge due to several reasons. FDA and EFSA heavily rely on research from the 1980s with Acceptable Daily Intakes (ADIs) that have not been updated. Most dyes are tested in isolation, leaving cumulative effects unassessed. Different countries have different policies for the regulation of synthetic dyes, which creates inconsistency and trade conflicts. Moreover, the older studies did not assess the neurobehavioral endpoints, further stressing the dire need to update the ADIs with recent data. Resistance from industry due to the reformulation costs, coupled with public unawareness and inadequate labeling, makes regulation more difficult.

These regulatory gaps must be addressed promptly by a public health approach that drives systemic change. Along with updating the FDA safety standards, public health campaigns like mass-media campaigns and school-based programs should be implemented to spread public awareness about the adverse neurologic effects of additive dyes, urging parents and children to read labels and choose dye-free alternatives or food with natural colorants since promoting natural food colorants and organic foods could eliminate the use of synthetic dyes, thus promoting a healthier society. Reformulation incentives could be provided to manufacturers for switching to natural colorants, along with the introduction of a tax/penalty for selling food high in additives to children. Point of sale warnings and transparent labeling can help society transition towards safer additive-free diets. Protecting the developing brains of children should be our utmost priority, for which we must choose safety over color. Our work is in line with the TITAN Guidelines on the need for transparency in AI use in healthcare[6].

## Data Availability

No data were generated for this manuscript.

## Peer and provenance statement

This article was not commissioned and was externally peer-reviewed.

## References

[1] Miller MD Steinmaus C Golub MS. Potential impacts of synthetic food dyes on activity and attention in children: a review of the human and animal evidence. Environ Health 2022;21:45. 35484553 10.1186/s12940-022-00849-9 PMC9052604

[2] Nigg JT Lewis K Edinger T. Meta-analysis of attention-deficit/hyperactivity disorder or attention-deficit/hyperactivity disorder symptoms, restriction diet, and synthetic food color additives. J Am Acad Child Adolesc Psychiatry 2012;51:86–97.e8. 22176942 10.1016/j.jaac.2011.10.015 PMC4321798

[3] Joute JR Chawhan P Rungsung S. Food additives and their associated health risks. Int J Vet Sci Anim Husb 2016;1:1–5.

[4] Schab DW Trinh NH. Do artificial food colors promote hyperactivity in children with hyperactive syndromes? A meta-analysis of double-blind placebo-controlled trials. J Dev Behav Pediatr 2004;25:423–34. 15613992 10.1097/00004703-200412000-00007

[5] Damotharan K Sudhakaran G Ramu M. Biochemical processes mediating neurotoxicity induced by synthetic food dyes: a review of current evidence. Chemosphere 2024;364:143295. 39260596 10.1016/j.chemosphere.2024.143295

[6] Agha RA Mathew G Rashid R. Transparency in the reporting of Artificial INtelligence – the TITAN guideline. Prem J Sci 2025;10:100082.

